# Extradural C1–C2 Dumbbell Schwannoma Mimicking an Intradural Lesion on MRI: A Case Report

**DOI:** 10.7759/cureus.116836

**Published:** 2026-09-24

**Authors:** Mamadou Bata Dianka, Yelka Matos Furones, Daoud Ali Mohamed, Luis F Gonzalez Vazquez, Yoniel Suarez-Guerrero, Heriberto Y Gonzalez Bonaga

**Affiliations:** 1 Neurosurgery, Hôpital Général Peltier, Djibouti City, DJI; 2 Neurology, North Georgia Clinical Research, Woodstock, USA; 3 Radiology, Hopital de la Police Nationale, Djibouti City, DJI; 4 Anesthesiology, Geisinger Medical Center, Danville, USA; 5 General Medicine, Asociacion Española de Socorros Mutuos, Montevideo, URY; 6 Internal Medicine, Crossover Health, Louisville, USA

**Keywords:** c1–c2 spinal lesion, cervical dumbbell schwannoma, cervical myelopathy, extradural tumor, spinal nerve sheath tumor

## Abstract

Cervical dumbbell schwannomas are benign nerve sheath tumors that may present diagnostic challenges when preoperative imaging suggests intradural involvement despite an extradural origin. We report the case of a 43‑year‑old African male with a three‑year history of right‑sided cervicobrachialgia and two years of progressive quadriparesis, sensory loss, and mixed reflex abnormalities consistent with cervical myelopathy. MRI demonstrated a 31 × 21 mm C1-C2 mass initially interpreted as intradural‑extramedullary. However, intraoperative findings revealed a purely extradural schwannoma arising from the nerve root, producing significant spinal cord compression through a classic dumbbell configuration. A posterior midline approach with C2 laminectomy allowed safe microsurgical resection, achieving gross total removal without cerebrospinal fluid leakage, without neurological deterioration, and with progressive improvement of quadriparesis. Postoperative MRI confirmed complete resection with expected inflammatory changes and no residual tumor. This case underscores the importance of correlating radiological and intraoperative findings, as cervical schwannomas may mimic intradural lesions on MRI. Accurate classification and tailored surgical planning are essential for optimal outcomes, particularly in resource‑limited settings.

## Introduction

Cervical spinal schwannomas represent 25%-30% of all spinal nerve sheath tumors [1], and dumbbell schwannomas constitute a distinct subgroup characterized by extension through the intervertebral foramen with combined intraspinal and extraspinal components [2]. These tumors account for 10%-15% of spinal schwannomas and are more frequently located in the cervical region due to the narrow canal and proximity to critical neurovascular structures [3]. Epidemiological studies report an incidence of 0.3-0.5 per 100,000 persons per year, with lower prevalence among Black populations, although underdiagnosis may contribute to variability [4,5]. Contrast‑enhanced MRI is the diagnostic modality of choice; however, distinguishing extradural from intradural components may be challenging. Early recognition of foraminal expansion and accurate classification are essential to prevent irreversible neurological deficits and guide surgical planning [6]. This article presents a case of a cervical dumbbell schwannoma initially interpreted as intradural on MRI but found to be purely extradural intraoperatively, highlighting the importance of radiologic-surgical correlation.

## Case presentation

A 43‑year‑old African male presented with a three‑year history of right‑sided cervicobrachialgia refractory to acetaminophen and ibuprofen. Two years prior to admission, he began to develop progressive functional impairment of all extremities. On clinical examination at presentation (Day 0), he exhibited quadriparesis with muscle strength graded 3/5, more pronounced in the right upper limb, and increased muscle tone throughout. Sensory testing revealed diminished tactile, thermal, and nociceptive sensation in the right hemibody. No sphincter disturbances were reported. Cranial nerve function was intact.

Imaging findings

An MRI performed on Day 1 revealed a right‑sided C1-C2 mass measuring 31 × 21 mm. The lesion appeared intradural-extramedullary on imaging, producing marked right‑lateralized spinal cord compression. A lobulated configuration with foraminal extension suggested a dumbbell schwannoma (Figure 1).

**Figure 1:**
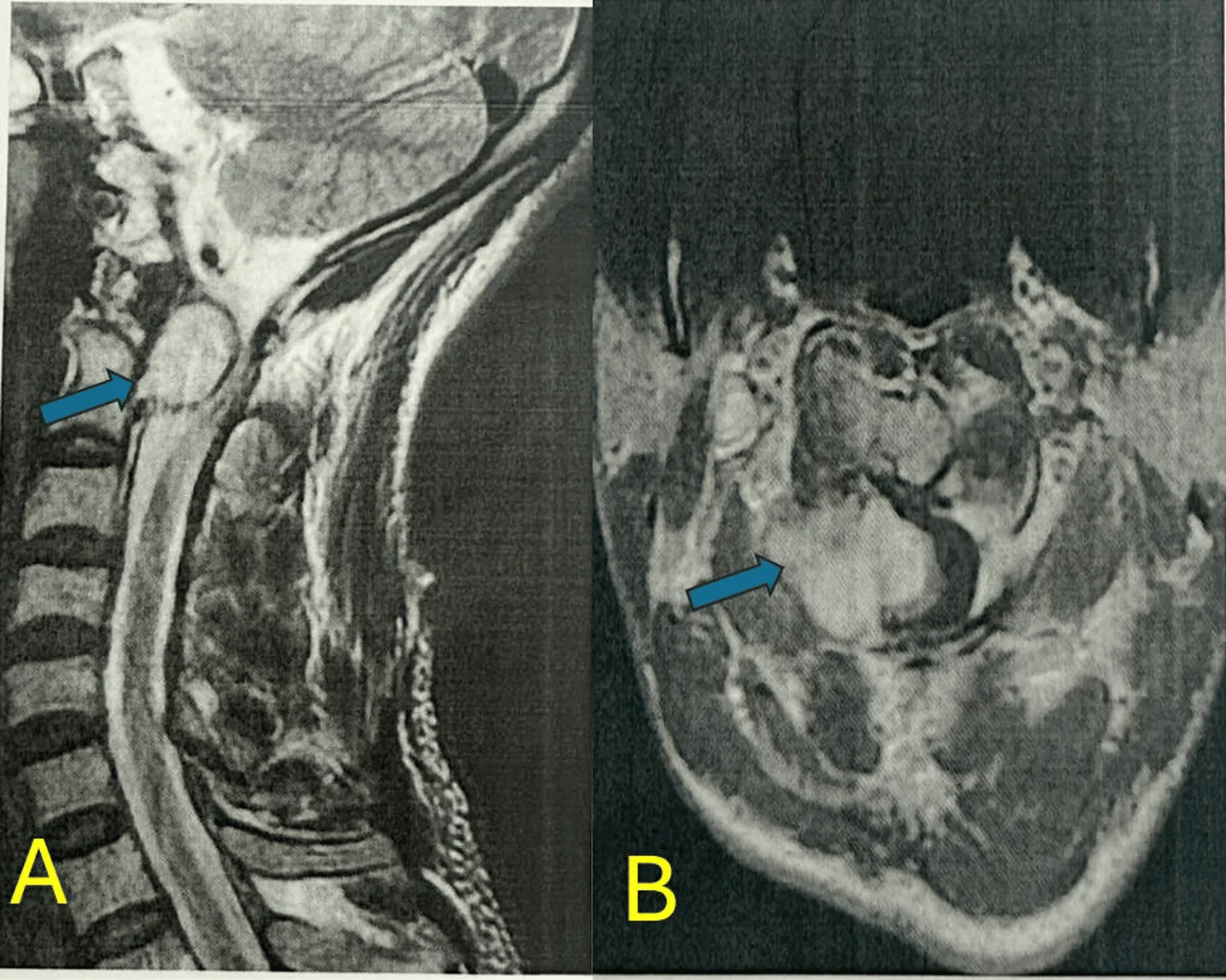
Preoperative MRI findings A) Sagittal T2‑weighted MRI sequence demonstrating a 31 × 21 mm extradural mass at the C1–C2 level, producing marked spinal cord compression. The lesion appears heterogeneously hyperintense on T2, and the blue arrow highlights the mass adjacent to the odontoid process and the C2 vertebral body. Mild T2 hyperintensity within the spinal cord is visible, consistent with compressive myelopathy. B) Axial T1‑weighted MRI with contrast showing an extradural lobulated soft‑tissue mass occupying the right side of the spinal canal at C2. The lesion is isointense on T1 and demonstrates strong, homogeneous gadolinium enhancement. The blue arrow indicates the tumor within the canal.

However, this radiological impression was later contradicted by intraoperative findings, which demonstrated a purely extradural tumor.

Preoperative workup

Preoperative laboratory evaluation is summarized in Table 1.

**Table 1:** Preoperative laboratory evaluation demonstrated values within normal limits for adult males.

Laboratory Test	Result	Reference Range	Units
White blood cell count	7,000	4,000–11,000	/µL
Hemoglobin	13	13.5–17.5	g/dL
Platelet count	2,00,100	150,000–450,000	/µL
Prothrombin time	13	11–15	seconds
Partial thromboplastin time	27	25–35	seconds
Blood type	O‑negative	-	-

Given the planned cervical spine surgery, the patient received cefazolin 1 g IV as a prophylactic antibiotic one hour prior to incision (Day 2).

Surgical intervention

On Day 2, a posterior midline approach from the occiput to C3 was performed. After C2 laminectomy, the dura appeared tense and bulging. The surgeon opened the dura to verify the presence of an intradural component; no intradural tumor was identified. The dura was immediately closed, and the procedure continued in an extradural fashion.

The schwannoma arose from a cervical nerve root and demonstrated soft consistency with mild venous engorgement. Extradural tumor removal was performed piecemeal, without the use of intraoperative neuromonitoring or an operating microscope. The involved nerve root was sacrificed to achieve complete resection. No cerebrospinal fluid leakage occurred. The dura was closed primarily, and the wound was closed in anatomical layers (Figure 2).

**Figure 2:**
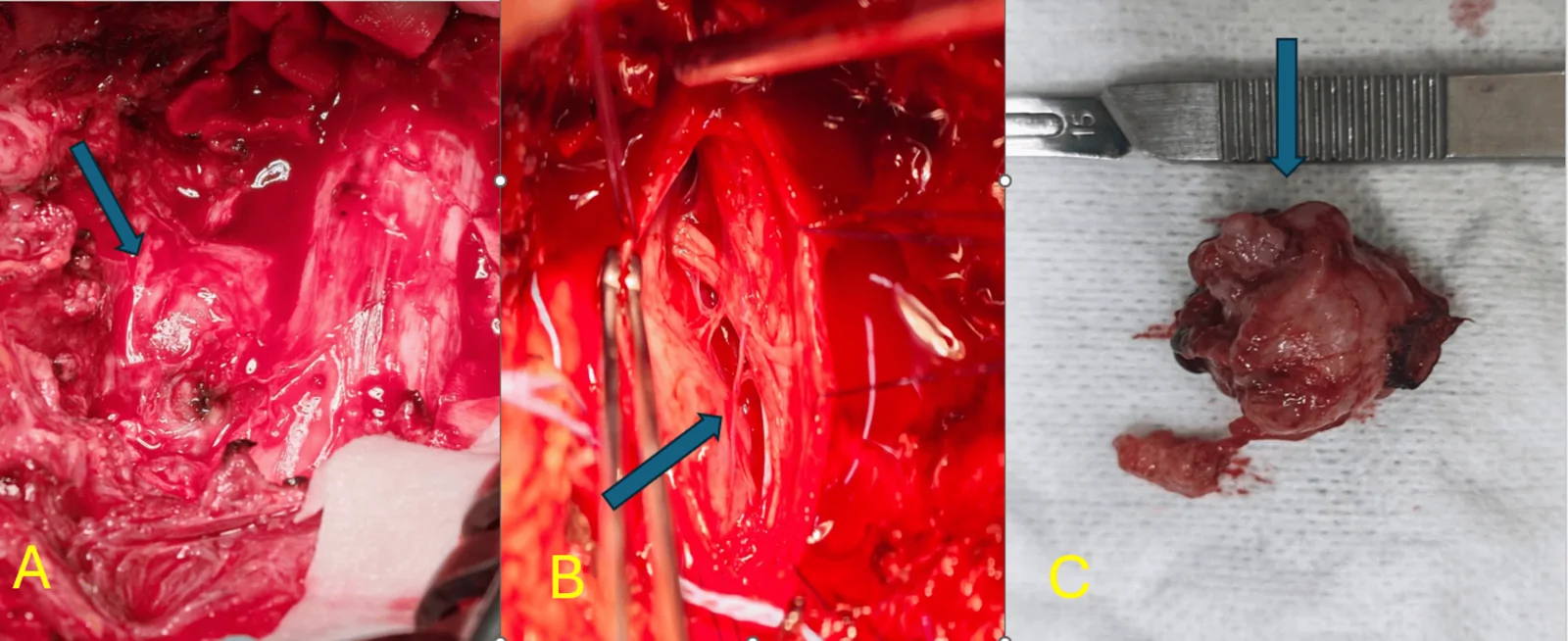
Intraoperative findings A) Extradural tumor adherent to the outer dural layer; a completely extradural schwannoma is visualized, firmly adherent to the external surface of the dura mater within the epidural space. The blue arrow indicates the tumor mass compressing the dura from the outside. B) Dural opening without intradural tumor; after opening the dura, no intradural tumor is present. The image shows the extradural mass protruding inward, deforming and compressing the dura mater from the epidural space. The blue arrow highlights the dural incision and the external compression caused by the tumor. C) The tumor was progressively debulked and removed in a piecemeal fashion. The blue arrow marks the schwannoma during the debulking process.

Histopathological confirmation

Findings confirmed a WHO Grade I schwannoma with benign spindle cell proliferation and storiform architecture (Figure 3) [7].

**Figure 3:**
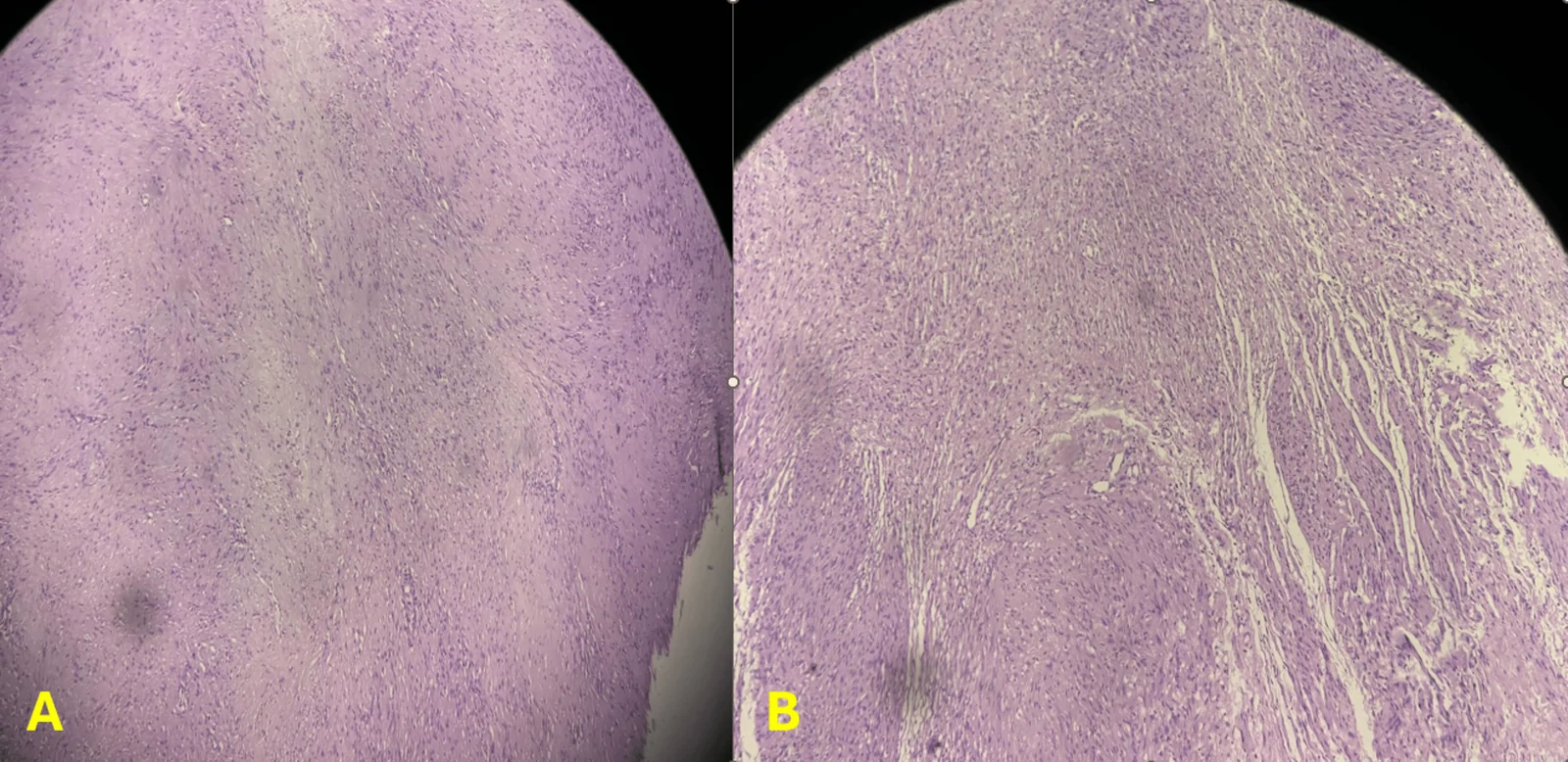
Histopathological findings A) Hematoxylin–eosin staining: Histological section showing a well‑circumscribed spindle‑cell proliferation surrounded by a thin fibrous capsule. The tumor is composed of schwannian spindle cells arranged in loose fascicles, with incipient Verocay body formation as described in the pathology report. No necrosis or significant mitotic activity is observed. B) Higher‑magnification view: The image demonstrates fusiform Schwann cells with wavy nuclei within an abundant myxoid–hyalinized stroma, consistent with a benign nerve sheath tumor. Ectatic vessels with thickened, hyalinized walls are present. No malignant features are identified.

Immediate postoperative course

On postoperative day 2, the patient remained clinically stable, with neurological function preserved relative to his preoperative status. His quadriparesis showed early improvement, particularly in right‑upper‑limb strength, and his preoperative cervicobrachialgia had significantly decreased. No new sensory deficits were noted, and there were no signs of cerebrospinal fluid leakage. Local wound care was performed, the drain was removed, and he continued to improve before discharge with follow‑up instructions.

Postoperative management

Postoperative treatment consisted of amoxicillin/clavulanic acid 1 g twice daily for eight days, following the institutional protocol used in this low‑resource regional setting to reduce postoperative wound infection risk. Analgesic management included paracetamol 1 g four times daily for ten days and subcutaneous morphine 10 mg three times daily for three days. Diazepam 10 mg nightly for five days was prescribed to assist with muscle relaxation and postoperative comfort. Local wound care was performed on postoperative days 1 and 2, and suture removal was scheduled for day 15. Active physiotherapy was recommended as tolerated.

Postoperative imaging

A postoperative cervical MRI obtained on Week 2 demonstrated complete surgical resection of the lesion with no residual tumor. The surgical bed showed expected postoperative changes, including localized edema and small areas of hematoma within the operative cavity, without mass effect or compression of neural structures. No cerebrospinal fluid collection or pseudomeningocele was identified (Figure 4).

**Figure 4:**
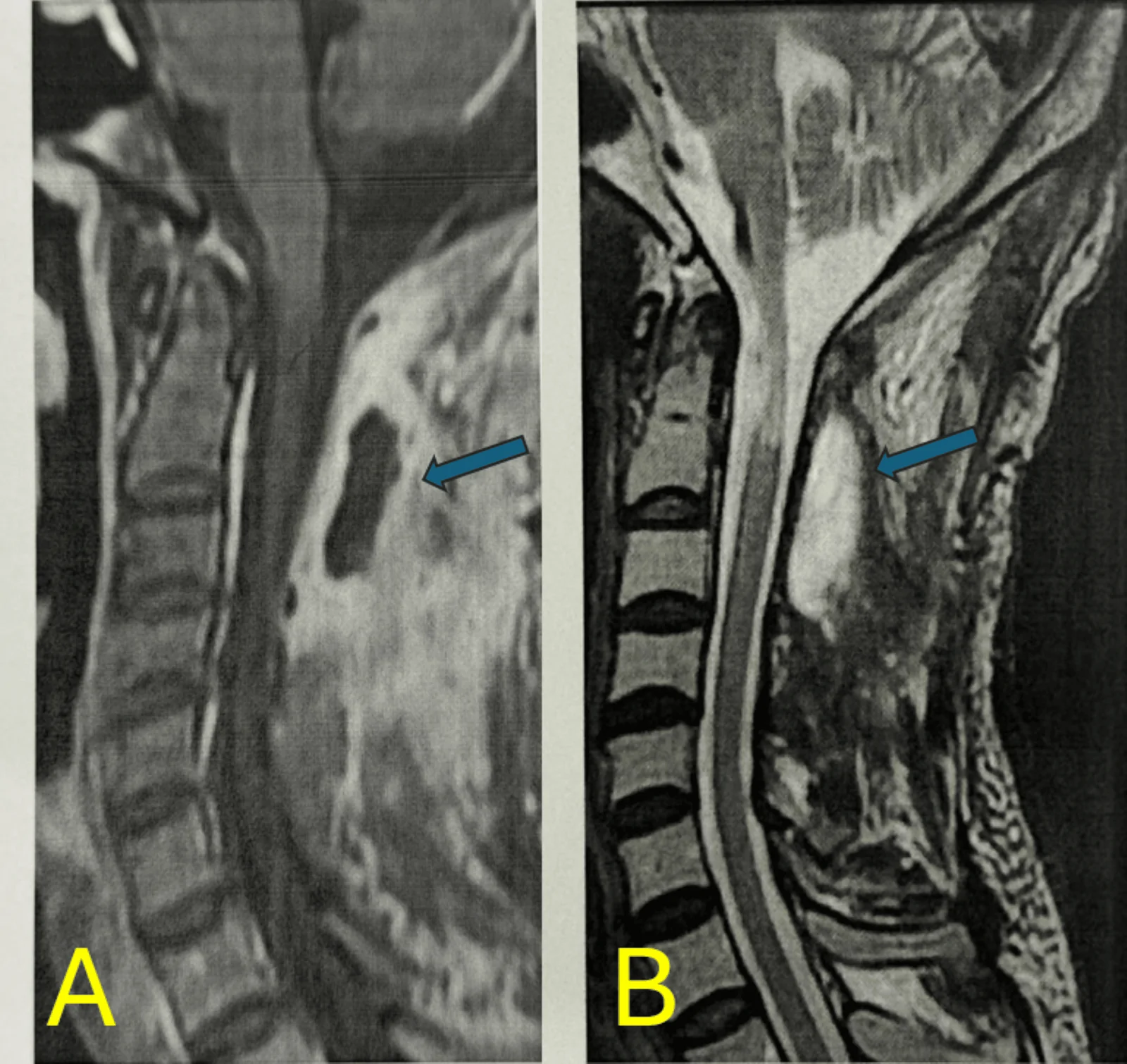
Postoperative MRI A) Postoperative sagittal T1-weighted MRI with gadolinium contrast demonstrating contrast enhancement at the surgical site, with surrounding postoperative edema. The blue arrow highlights the region of complete resection, with no residual tumor identified. B) Postoperative sagittal T2-weighted MRI showing complete excision of the lesion. The surgical bed demonstrates T2 hyperintensity compatible with postoperative inflammatory changes, with no evidence of residual mass. The blue arrow indicates the postoperative cavity.

Follow‑up

At the one-month follow‑up visit, the neurosurgical team documented favorable clinical evolution and authorized medical discharge. The patient continued regular follow‑up, and at the three‑month visit, he demonstrated the neurological improvement described below: He was able to walk slowly without assistance, with full muscle strength (5/5) throughout the left hemibody, 4/5 strength in the right upper limb, and 5/5 in the right lower limb.

## Discussion

The present case illustrates a well‑documented but still challenging phenomenon in spinal tumor surgery: the discrepancy between preoperative MRI interpretation and intraoperative reality. Although MRI remains the gold standard for evaluating spinal nerve sheath tumors, several studies have shown that extradural schwannomas may mimic intradural-extramedullary lesions, particularly in the cervical region where the canal is narrow and the dura is easily deformed by mass effect [1-3]. This radiologic-surgical discordance has been described across multiple epidemiologic cohorts, including populations with limited access to advanced imaging, where delayed diagnosis may further complicate interpretation [4].

In our patient, preoperative MRI suggested an intradural‑extramedullary mass at C1-C2. The lobulated contour, prominent foraminal extension, and marked compression of the spinal cord were consistent with a dumbbell schwannoma. Although intraoperative exploration revealed a purely extradural tumor, it still met the anatomical criteria for a dumbbell configuration, as the lesion extended through the neural foramen without an intradural component. Similar cases have been reported in the literature, where dural compression, effacement of the subarachnoid space, and distortion of the cord create the false appearance of intradural origin on MRI [5,6]. These findings highlight the limitations of imaging in complex cervical lesions and reinforce the principle that the definitive anatomical diagnosis is established in the operating room.

Radiologically, distinguishing schwannomas from other intradural‑extramedullary tumors is essential for surgical planning. Schwannomas typically demonstrate T2 hyperintensity and foraminal widening, whereas meningiomas often show homogeneous enhancement and a dural tail [8]. An additional helpful feature is the absence of a cerebrospinal fluid cleft (“dural sign”), which is often present in intradural‑extramedullary tumors but absent in extradural lesions such as this one. Neurofibromas may mimic schwannomas but frequently exhibit fusiform nerve involvement and lack a well‑encapsulated appearance. Paragangliomas, although rare, display intense vascularity and flow voids. Infectious, vascular, and post‑traumatic lesions can also simulate extradural tumors but usually present with systemic symptoms or distinct flow characteristics. In this case, the imaging pattern-combined with the absence of systemic signs and the histopathological confirmation-supported the diagnosis of schwannoma and excluded alternative etiologies [9-11].

From a surgical perspective, dumbbell schwannomas require careful planning due to their foraminal extension and proximity to the vertebral artery. The Yang et al. classification provides a reproducible framework for determining the optimal approach based on dominant tumor location and vertebral artery involvement [5]. Our lesion corresponded to a Type B (foraminal‑dominant) dumbbell schwannoma, for which a posterior midline approach with C2 laminectomy is recommended. Although the preoperative assumption of intradural involvement suggested the need for intradural dissection, the intraoperative finding of a purely extradural tumor simplified the procedure and reduced the risk of cerebrospinal fluid leakage.

Large surgical series have demonstrated excellent outcomes following complete resection of cervical schwannomas, with low recurrence rates when gross total removal is achieved [2,3]. Our patient experienced early neurological improvement, consistent with previously reported cases, including African cohorts where extradural schwannomas, though less common, show comparable surgical outcomes when managed with meticulous microsurgical technique [6,11].

Ultimately, this case underscores a fundamental truth in neurosurgery: imaging guides the surgeon, but the final diagnosis is made in the operating room and confirmed by histopathology. The process-from radiologic suspicion to intraoperative discovery and pathological confirmation-reflects the complexity and beauty of medical practice, where multiple steps converge to reveal the true nature of a lesion. Even with modern imaging, the surgeon must remain prepared for unexpected findings, adapting the operative strategy to ensure safe and effective tumor removal.

Limitations

This report describes a single case, limiting generalizability. Although imaging suggested intradural involvement, intraoperative findings confirmed an extradural origin, illustrating the limitations of MRI in complex cervical lesions. Advanced neuroimaging or intraoperative ultrasound might have improved preoperative characterization. Long‑term follow‑up is pending to assess recurrence risk.

## Conclusions

Cervical dumbbell schwannomas may appear intradural on MRI due to marked dural compression, yet definitive characterization is often established intraoperatively. In this case, surgical exploration revealed a purely extradural lesion despite imaging suggesting intradural involvement. This underscores the importance of correlating radiological impressions with operative findings and histopathology. A posterior midline approach enabled complete resection and neurological improvement, highlighting the value of meticulous microsurgical technique, especially in resource‑limited settings.
